# Intravenous insulin protocol reduces time to target glucose in critically ill trauma and burn patients

**DOI:** 10.62838/jccm-2026-0027

**Published:** 2026-07-27

**Authors:** Oluwafolaranmi E. Sodade, Connor J. English, Cindy L. Austin, Charles A. Lunday, Yang Wang, Brian B. Draper

**Affiliations:** Trauma and Burn Research, Mercy Hospital, Springfield, Missouri, USA; Kirksville College of Osteopathic Medicine, Kirksville, Missouri, USA; Clinical Pharmacy, Mercy Hospital, Springfield Missouri, USA; Enterprise Analytics, Mercy Health, East Chesterfield, Missouri, USA; General and Trauma Surgery, Mercy Clinic, Springfield, Missouri, USA

**Keywords:** hyperglycemia, hypoglycemia, glucose variability, stress induced hyperglycemia, intravenous insulin, subcutaneous insulin, intravenous insulin protocol, blood glucose titration, tight glycemic control, intensive insulin therapy

## Abstract

**Introduction:**

Glycemic control is vital in the management of critically ill patients. Scientific evidence has proven that a drastic change in blood glucose levels can lead to adverse outcomes including increased hospital, ICU length of stay, morbidity, and mortality. Despite the challenges in developing standardized intravenous insulin protocols, institutions have successfully implemented protocols in various settings. Our tertiary care hospital has an established intravenous insulin protocol for the cardiac ICU. Given the complexity of managing critically ill patients, an intravenous insulin protocol for such patients was implemented in February 2022. The study aimed to evaluate the effectiveness of the newly implemented intravenous insulin protocol in glycemic control of critically ill trauma and burn patients.

**Methods:**

A single center retrospective chart review was conducted on 230 patients: 119 patients were extracted from the pre-protocol implementation and 111 patients post-protocol implementation periods. Ninety-nine patients were excluded due to on-admission diagnosis of diabetic ketoacidosis, hyperosmolar hyperglycemic state, or incomplete data. Data collection included: type of injury; body mass index, pre-existing comorbidities; insulin administration times; target glucose actualization; glycemic events; hospital and ICU length of stay, mortality rates in the ICU, hospital, and 30 days post-hospitalization.

**Results:**

In the post-implementation group, the time to reach target glucose was significantly reduced when compared to the pre-implementation group. The rates of glycemic events after achieving target glucose were similar with a slightly lower rate post-implementation. There were no differences in the length of stay or mortality rates during hospitalization or 30-days post-hospitalization between the groups. However, when comparing routes of insulin administrations, the intravenous insulin significantly showed better glucose control and reduced the rates of glycemic events than the subcutaneous route.

**Conclusion:**

Intravenous insulin protocol demonstrated a significant reduction in the time to target glucose levels for the critically ill patients.

## Introduction

Hyperglycemia is common in critically ill patients, even those without pre-existing diabetes. Factors like stress, acute illnesses, injuries, burns, and infections can raise blood glucose (BG) levels in previously glucose-tolerant individuals [1].

In clinical practice, several factors must be considered in the management of critically ill patients, which include BG targets, history of diabetes mellitus, other comorbidities, available technology, provider’s workload, monitoring equipment and the route of insulin administration [2].

Commonly, hyperglycemia observed in critically ill patients is an adaptive response to stress. Neurocritical ill patients with acute brain injury are more prone to glycemic changes. This is because of the brain continuous dependence on glucose. Hyperglycemia, which is most common in such patients have been associated with adverse outcomes such as ischemic stroke, intracranial and subarachnoid hemorrhage [3,4,5].

Burn patients are considered to have hypermetabolic needs with responses involving several pathways including glucose, leading to insulin resistance and hyperglycemia which affects post-burn outcomes. Severely burned patients with poorly controlled glucose are susceptible to increased incidence of bacteremia, fungemia, enhanced catabolism and mortality [6].

Several observational studies have shown increases in morbidity and mortality in critically ill patients with hyperglycemia irrespective of their diabetes status [4]. Although hyperglycemia during a critical illness in diabetics is understood to pose danger, patients not previously known to have diabetes are at a higher risk of hyperglycemia. Studies have shown that patients who were not previously known diabetics suffered a greater mortality rate and a lengthier hospital stay than their known diabetic counterparts. Interestingly, outcomes in individuals with stress-induced hyperglycemia experienced worse outcomes than critically ill hyperglycemic patients with pre-existing diabetes [4,5,6].

Early hyperglycemic control is an important step in the management of critically injured trauma patients. Hyperglycemia within the first week post traumatic event was associated with significantly greater hospital and intensive care unit (ICU) length of stay (LOS), ventilator time, infection, and mortality [7]. Due to the relatively high rates of hyperglycemia in critically ill patients, increased mortality, adverse events, and general clinical outcomes, the American Diabetes Association (ADA) [8] and the American Association of Clinical Endocrinologists [9] recommended the use of continuous intravenous (IV) insulin.

Our hospital has an existing insulin infusion protocol (IIP) that is utilized in the cardiac ICU. This study will evaluate use of a newly implemented IIP for the management of critically ill trauma and burn patients.

Based on existing literature and the recommendations of the ADA, we hypothesized that BG control by an IIP would show better glycemic control and improved clinical outcomes in comparison to critically ill trauma and burn patients who were managed without an IIP.

The objective of the study is to compare various aspects related to glucose control using an IIP versus a non-IIP in critically ill trauma and burn patients.

Per our institutional IIP guidelines, IV insulin was initiated when there were two blood glucose consecutive readings greater than > 200, while targeting a BG range of 140–180. The time to target BG was recorded as the time between IV insulin was initiated to the time the target glucose range of 140–180 was actualized. A glucose event is recorded when BG is over 180 (Hyperglycemia BG >180) or less than 70 (Hypoglycemia BG <70) after already achieving target glucose.

Subsequent glucose events were recorded as a single glucose event (regardless of the numbers of consecutive BG readings), that falls out of the normal glycemic range of (70–180) up to seven days of hospital admission.

The time to consistent BG control was defined as the time between IV insulin initiation until the time the first glucose reading falls within the target range.

We monitored and recorded BG every four hours during subQ insulin administration and following the recommendations of the ADA and the Society of Critical Care Medicine, BG were monitored and recorded every hour during IV insulin administration. According to Lal et. al, the frequency of BG monitoring does not significantly reduce the risk of hypoglycemic episodes [10].

## Materials and methods

A single center, two-arm-combined, observational, and retrospective chart review was approved as exempt by the Institutional Review Board. The study included adult (≥ 18 years old), male and female trauma or burn patients admitted to the neuro trauma ICU or Burn Unit of our tertiary care hospital who required insulin therapy for glycemic control. Patients with an active diagnosis of diabetic ketoacidosis or hyperglycemic hyperosmolar state during index hospitalization were excluded.

### Inclusion criteria

- Adult (≥ 18 years old), male and female trauma and burn patients
- Admitted to Mercy Hospital Springfield 4E Neuro Trauma Intensive Care Unit (NTICU) or 7H Burn Unit
- Required insulin therapy

### Exclusion criteria

Patients with an active diagnosis of Diabetic Ketoacidosis (DKA) or Hyperglycemic Hyperosmolar State (HHS) during index hospitalization

Data was collected from electronic medical record (EMR) via a chart review for both groups of the study. The period of data collection for the pre-implementation group was March 2020 until January 31, 2022, while that of the post-implementation group was from February 1, 2022, until the target number of enrollment was achieved.

**Group A:** (non-IIP group/pre-protocol implementation group): Patients admitted to the Neuro-trauma ICU or Burn Unit and managed for glycemic control between March 2020 until January 31, 2022.

(Male 52, Female 15 = 67) (Male: Caucasian= 43, African American =1, Other Race =8), (Female: Caucasian=13, Other Race=2). Average Age = 54.7.

The mechanisms of injury in this group: (Trauma=46, Burn =17, Infections =4).

**Group B:** (IIP group/post-protocol implementation): Patients admitted to the Neuro-trauma ICU or Burn Unit and managed for glycemic control from February 1, 2022, until target enrolment was achieved. (Male 43, Female 21=64) (Male: Caucasian= 39, African American =1, Other Race =3), (Female: Caucasian=19, Other Race=2). Average age = 55.04.

The mechanisms of injury in this group: (Trauma=21, Burn =8, Infections =13, Others= 22).

Data elements collected included the date of admission to 30 days following hospital discharge, age, sex, race, obesity (BMI > 30 kg/m^2^), and other comorbid conditions (e.g. diabetes, hypertension, kidney disease). Additionally, the type of injury (trauma or burn), in-hospital mortality, and 30-day mortality were recorded. Insulin-specific measures included initial receipt of subcutaneous (subQ) insulin, time to initiation of IV insulin, and time of transition from IV insulin back to subQ insulin.

Protocol adherence was determined by identifying whether IV insulin was initiated following two consecutive BG levels at 200 mg/dL and above, the threshold value for initiation of the protocol. Hyperglycemia was defined as BG levels higher than 180mg/dL, and hypoglycemia was BG levels lower than 70mg/dL.

The primary outcomes included time to consistent BG control in hours, which was defined as two consecutive glucose measurements in the target range following initiation of the protocol, number of hyperglycemic events (BG > 180 mg/dL) after target glucose is achieved during the first seven days of admission, and number of hypoglycemic events (BG < 70 mg/dL).

Secondary outcomes include length of ICU stay and length of hospital stay in days, ICU mortality, inhospital mortality, and 30-day mortality. Summary of cost-effectiveness of IV insulin administration in comparison to subcutaneous insulin and adherence to the IIP were descriptively analyzed.

Weighted regression analysis was then applied to evaluate the IIP effect on the outcomes and Statistical significance was assessed with a threshold of 0.05.

## Results

We assessed the effects of the newly implemented IIP on trauma and burn patients by comparing the performance of the pre- and post-protocol implementation groups across the following key outcomes. Additionally, we evaluated the impact of introducing IV insulin during the post-implementation period by comparing its effectiveness with subQ insulin administration across both the pre- and post-implementation protocol periods.

### Outcome 1: Time to target glucose.

**Table 1** (second column) and **Figure 1** illustrate the impact of the IIP on the time to reach target glucose levels. The IIP significantly reduced the time to target glucose (10.06 ± 5.69 hours vs 24.96 ± 34.59 hours for pre-implementation group). A propensity score weighting approach was employed to balance covariates between the two groups, including demographic variables, type of injury, and pre-existing comorbidities. This technique reduced the confounding bias and ensured a fairer comparison by making the groups more comparable on those observed characteristics. The result indicated that the IIP significantly affects the time to reach the target glucose level (*p* < 0.0001).

**Fig. 1.**
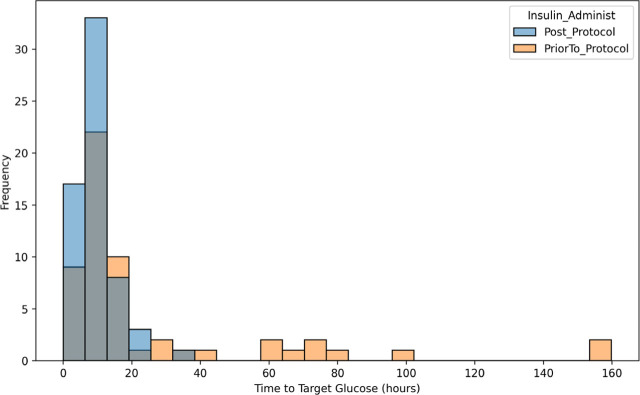
Time to Target Glucose in the pre- and post-implementation of Intravenous Insulin Protocol.

**Table 1.** Comparison of Mean and Standard Deviation of the Outcomes Pre (Non-IIP group) and Post-Implementation of IIP (IIP group)

**Insulin Protocol**	**Time to Target Glucose (SD)**	**Hyperglycemia rate within 7 days of Admission**	**Hypoglycemia rate within 7 days of Admission**	**Hospital LOS**	**ICU LOS**
Post-Implementation (IIP)	10.06 (5.69)	56.1% (0.30)	0.4% (0.1)	17.09 (18.67)	11.97(18.48)
Pre-Implementation (Non-IIP)	24.96 (34.59)	45.3% (0.28)	0.8% (0.8)	19.81 (21.55)	13.84(18.37)

### Outcome 2: Occurrences of hyperglycemic and hypoglycemic events within 7 days of hospital admission

**Table 1** (column 3) and **Figure 2** show the impact of the IIP on reducing hyperglycemia (BG >180 mg/dL) during the first seven days of hospital admission. Patients treated during the pre-implementation period had a slightly lower mean percentage of hyperglycemic events (45.3%) compared to those treated after protocol implementation (56.1%). The propensity score weighting, and regression analysis indicated no significant effect on hyperglycemia control (*p* = 0.193).

**Fig. 2.**
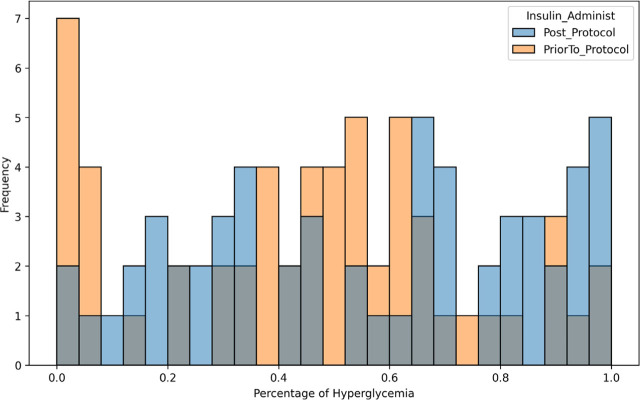
Percentage of Hyperglycemia in the pre-and post-implementation of Intravenous Insulin Protocol.

**Fig. 3.**
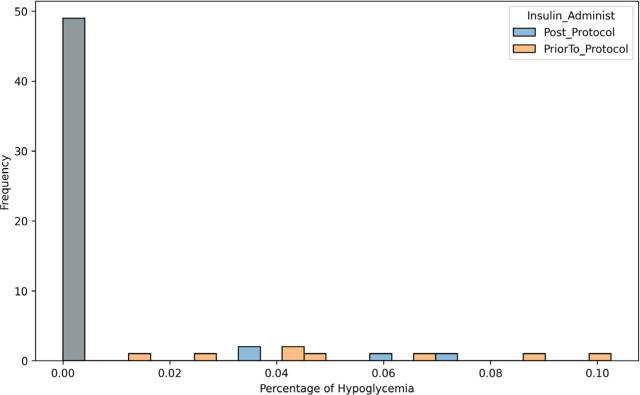
Percentage of Hypoglycemia in the pre- and post-implementation of Intravenous Insulin Protocol.

The 4th column in **Table 1** and **Figure 3** examined the occurrence of hypoglycemia, defined as BG levels below 70, within the same period. Post-protocol implementation group showed a slightly lower mean and standard deviation, but this reduction is not sufficient to demonstrate the protocol’s superiority in reducing hypoglycemia. The weighted regression analysis indicated that the IIP does not significantly impact hypoglycemia management (*p* = 0.588).

### Outcome 3: Occurrences of hyperglycemia and hypoglycemia during the post-protocol implementation period

**Table 2** shows the percentage of hyperglycemia and hypoglycemia events during IV and subQ insulin administration in the post-protocol period. Hyperglycemia was significantly lower during IV insulin administration, with a mean of 9.8% compared to 56.1% during subQ insulin administration. In addition, hypoglycemia was better controlled with IV insulin, with a mean of 0.2% versus 0.4% for subQ insulin.

**Table 2.** Comparison of Mean and Standard Deviation Percentages of Hyperglycemia and Hypoglycemia During Intravenous and Subcutaneous Insulin Administrations Before and After Protocol Implementation

**Insulin Route of Administrations**	**Hyperglycemic events (SD)**	**Hypoglycemic events (SD)**
IV Insulin (Post-Implementation)	9.8 % (0.14)	0.2% (0.02)
SubQ Insulin (Post-Implementation)	56.1 % (0.3)	0.4% (0.01)
SubQ Insulin (Pre-Implementation	45.3% (0.28)	0.8% (0.02)

A paired sample sign test confirmed the statistical significance of these differences, with *p*-values for both hyperglycemia and hypoglycemia reduction being less than 0.0001. This indicated that IV insulin administration is more effective in controlling both hyperglycemia and hypoglycemia in trauma and burn patients.

### Outcome 4: Occurrences of hyperglycemia and hypoglycemia during subQ insulin administration in the pre-implementation group versus IV insulin administration in the post-implementation group

The first and third rows in **Table 2** compared the mean percentages of hyperglycemia and hypoglycemia events during the IV insulin administration after protocol implementation and subQ insulin administration prior to protocol implementation. The mean percentage of hyperglycemia decreased significantly, from 45.3% during subQ insulin administration to 9.8% during IV insulin administration. For hypoglycemia, IV insulin also showed better control, with a mean of 0.2% compared to 0.8% for subQ insulin.

Using propensity score weighting and weighted regression analysis, the intervention’s effect on hyperglycemia was statistically significant (*p* < 0.0001), indicating a strong reduction in hyperglycemic events. However, the effect on hypoglycemia was not significant (*p* = 0.133), suggesting no substantial change in hypoglycemic events. Overall, IV insulin was highly effective at reducing hyperglycemia in trauma and burn patients.

### Outcome 5: Hospital and ICU LOS

The last two columns of **Table 1** compare the impact of the IIP on the LOS in the hospital and ICU. After IIP implementation, the mean hospital LOS was 17.09 ± 18.67 days, and the mean ICU LOS was 11.97 ± 18.48 days, compared to 19.81 ± 21.55 days and 13.84 ± 18.37 days before implementation. Propensity score weighting and regression analysis resulted in *p*-values of 0.49 for ICU stay and 0.102 for hospital stay, indicating that the IIP had no significant effect on the LOS in either setting.

### Outcome 6: Mortality rates

**Table 3** illustrates the mortality rates associated with the implementation of the IIP during hospital stay, in the ICU, and within 30 days post-hospitalization. Patients treated after the protocol implementation had lower mortality rates in the ICU (12.5% vs 16.9%) and within 30 days post-hospitalization (18.8% vs 23.7%) compared to the pre-implementation group. However, hospital mortality was slightly higher in the post-implementation group (18.8% vs 18.6%).

**Table 3.** Mortality rates in hospital, ICU, and 30 days post-hospitalization before and after implementation of the insulin protocol

**Insulin Protocol**	**Hospital**	**ICU**	**30 days post-hospitalization**
Post-Implementation (IIP)	18.8 %	12.5%	18.8%
Pre-Implementation	18.6%	16.9%	23.7%

To make a more reliable evaluation of treatment effect, propensity score weighting with weighted logistic regression analysis was employed to examine the effects of implementation of IIP on mortality rates. The results, with *p*-values of 0.487 for ICU mortality, 0.988 for hospital mortality, and 0.50 for 30-day post-hospitalization mortality, indicated that the IIP does not significantly affect mortality rates in the ICU, hospital, and 30-day post-hospitalization settings.

## Discussion

The newly implemented IIP significantly reduced the time to target glucose and showed potential for decreased glucose variability (GV) levels in the management of critically ill patients when compared to the pre-implementation group. Similarly, the prevalence of glycemic events was reduced during IV insulin administrations in the post-implementation group, when compared to subQ insulin administrations in both groups. This is consistent with multiple studies, that have demonstrated and recommended intensive insulin therapy and IV insulin administration as the preferred route of administration to effective glycemic control in critically ill adults [11,12,13,14,15,16].

Notwithstanding the years of research, intensive insulin therapy does not appear to benefit all critically ill patients as conversations regarding the specifics of standardized IIPs across departments and institutions continued to emerge [17].

Kramer et. al, conducted a systematic review and meta-analysis of randomized controlled trials involving 1248 neuro-critical patients. The study outcomes suggested that intensive insulin therapy with very loose glycemic control increases the risk of hypoglycemia, worsened neurological recovery and does not reduce mortality rates [18].

Hypoglycemia is another predictor of poor clinical outcomes in both critically and non-critically ill patients, such that a single episode of severe hypoglycemia can independently increase the risk of mortality. Safe implementation of tight glycemic control (TGC) requires appropriate monitoring to reduce the risk of this complication.

TGC is defined as BG between 80–110 mg/dL in continuous insulin infusion regimens and has been studied extensively in critically ill patients to improve clinical outcomes. However, factors such as BG targets in critical care patients, optimal glycemic targets for inpatients, the resources needed to attain tight TGC, education of service providers, adherence to protocols, accuracy in BG monitoring technologies, titration in rapidly changing glucose levels, modalities of insulin administration and GV are largely determinant of patients’ overall outcomes [18,19,20,21,22].

GV is defined as the fluctuation of BG or other glucose homeostasis over a given time. The increased GV across the heterogeneous critical care population (diabetics, non-diabetics, and stress-induced hyperglycemic patients) has been linked to increase risk of infectious morbidity, higher LOS, and therefore, informed the calls for routine administration of IV insulin to normalize glucose levels. Strategies to maintain optimal glycemic control in many critical care-IIPs have been conflicting. Multiple clinical studies have suggested varying target glucose range from 80–110 mg/dL to <200 mg/dL [22]. None of these studies, however, have significantly proven to determine the most efficient target glucose range in critically ill patients. Alternatively, a close range of BG targets between 140 to180 mg/dL have been suggested for glycemic control and reduction of hypoglycemic episodes with an overall increase of survival [23].

Our institutional IIP initiated IV insulin to target glucose between 140–180 mg/dL after two consecutive BG readings of >200 mg/dL. Once target glucose was achieved, the continuous IV insulin was switched to a maintenance subQ insulin and monitored closely. To this study, glucose readings were recorded up to seven days of admission. Outcome one showed that the post-implementation group, otherwise known as the IIP group, had a significantly reduced time to target glucose when compared to the pre-implementation (non-IIP) arm (10.06 ± 5.69 hours vs 24.96 ± 34.59 hours). This substantial difference was statistically significant (t = −2.5, p = 0.0076), supporting the alternative hypothesis that the IIP insulin reaches target glucose more quickly than the non-IIP group. One of the several limitations of this study is the overlap between the periods prior to protocol implementation to the time of protocol implementation, which led to the inability to determine if the IIP group had a significantly reduced GV when compared to the non-IIP group. However, the markedly larger standard deviation in the subQ insulin administration group, the greater GV and less predictable response compared with the more consistent trajectory observed with IV insulin is an indicator that the IIP has a potential of a reduced GV in comparison to the non IIP group.

Outcome two showed that patients treated during the pre-implementation period had a slightly lower mean percentage of hyperglycemic events (45.3%) compared to those treated after protocol implementation (56.1%). The non-IIP group of patients had a lower hyperglycemic event compared to the IIP group. On the other hand, the IIP group showed a slightly lower mean and standard deviation, but this reduction is not sufficient to demonstrate the protocol’s superiority in reducing hypoglycemia. The weighted regression analysis indicated that the IIP does not significantly impact hypoglycemia management (*p* = 0.588).

Outcome three indicated a significant reduction of glycemic episodes during IV administration. A paired sample sign test confirmed the statistical significance of these differences, with *p*-values for both hyperglycemia and hypoglycemia reduction being less than 0.0001. This further validates existing literature [11,12,13,14,15,16] and the recommendation of the society of critical care medicine guidelines on glycemic control for critically ill children and adults. Preference for an insulin infusion for the acute management of hyperglycemia with titration, guided by an explicit clinical support tool of frequency (≤ 1 hour) monitoring intervals during glycemic instability, was determined to minimize hypoglycemia. These recommendations are intended for consideration within the framework of the patient’s existing clinical status. Further research is required to evaluate the role of individualized glycemic targets, continuous glucose monitoring systems, explicit decision support tools, and standardized glycemic control metrics [24].

Outcomes four, five and six showed that there were no significant differences in the number of glycemic events, LOS either in the ICU or hospital, and mortality rates in the ICU, hospital and after the 30-day post-hospital discharge, when the pre- and post-implementation groups were compared. This could be associated with varying factors which includes the overlap between the pre- and post-implementation periods, measurement errors, estimation biases during transitioning of IV insulin to subQ insulin after target glucose was achieved.

We acknowledge several limitations of this study, which includes single-center study design, the heterogeneity of the patients’ populations, and the non-classification of the impact of the IIP on each mechanism of injuries-Trauma, Burn, Infection, indicating that our results may have applied differently if separated.

Other factors which could have considerably affected the clinical outcomes such as the ISS of individual patients, the nutritional supplementation, the use of corticosteroids and vasoactive medications were beyond the scope of this study.

Additionally, overall costs of protocol implementation ranging from service provisions, renumerations and costs of insulin administration were not calculated in determining the actual savings post-implementation of the IIP.

Prolonged illness in patients leads to increased mortality rates, with most deaths attributed to sepsis and multisystem organ failure. Maintaining normoglycemia with insulin in critically ill patients is vital to improving neurologic, cardiovascular, and infectious outcomes. Several studies have established a significant reduction in morbidity and mortality with aggressive insulin therapy in different clinical settings [25,26]. This study was unable to prove the survival benefits of our newly implemented IIP over the non-IIP group.

Despite recording a 100 percent compliance by physicians in putting orders for IV insulin administration following two consecutive BG levels of 200 mg/dL and above, we couldn’t determine adherence to the full implementation of the IIP. This could be attributed to the user-friendliness and general acceptability of the IIP, education of the care team and BG monitoring technologies and titration in rapidly changing glucose levels.

Notably, the successes of the IIPs depend largely on appropriate BG monitoring and titration. Studies have shown that a computerized insulin IV protocol, a continuous BG sensor, electronic glycemic management systems and an FDA approved insulin-dosing calculator can achieve tighter glycemic control without increasing hypoglycemia and mortality rates [19, 26,27,28].

A work by Clergeau et. al, concluded that a well-accepted, dynamic, individualized and easy to implement paper-based protocol can be an alternative to standard computerized systems [29]. Based on this information, there are needs to evaluate in subsequent studies, the role of individualized glycemic targets, continuous glucose monitoring, standardized glycemic control metrics and satisfaction survey to determine acceptability for IIP modifications and improvements.

## Conclusion

In conclusion, the IIP demonstrated a statistically significant impact on reducing the time to target glucose levels for trauma and burn patients and showed great potential in GV reduction. Equally, our study corroborates multiple studies on IV insulin as the preferred route of administration to effectively control the occurrence of glycemic events in critically ill patients. There is, however, room for improvement as regards protocol adherence, acceptability, technology, nursing support, care-team education, feedback surveys to enhance protocol modifications and improving overall clinical outcomes. Further studies with multiple ICU centers, larger sample size and specific patients’ data – ISS, comorbidities, nutritional supplementation, the use of corticosteroids and vasoactive medications are required to confirm our findings and to determine IIP cost-effectiveness, compliance, and to elaborate on the potentials in reducing complications in critically ill patients.
